# Supplementary material for: DNA nanopores as artificial membrane channels for bioprotonics
Source: Nat Commun. 2023 Sep 4;14:5364. doi: 10.1038/s41467-023-40870-1 (PMC10477224; doi:10.1038/s41467-023-40870-1)
Supplement: Supplementary file 1 — Supplementary Information [file 41467_2023_40870_MOESM1_ESM.pdf]

DNA nanopores as artificial membrane channels for DNA-based bioelectronics

Le Luo, Swathi Manda, Yunjeong Park, Busra Demir, Jesse Sanchez, M.P. Anantram, Ersin Emre Oren, Ashwin Gopinath†, and Marco Rolandi†

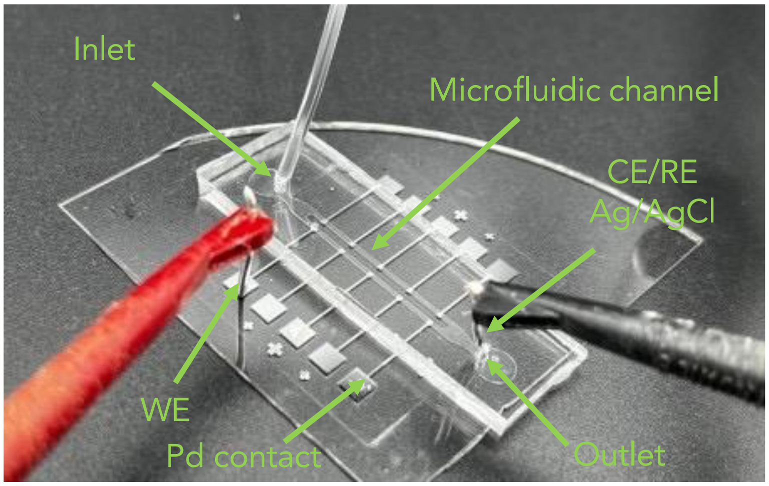

Supplementary Fig. 1 Optical image of the bioprotonic device.

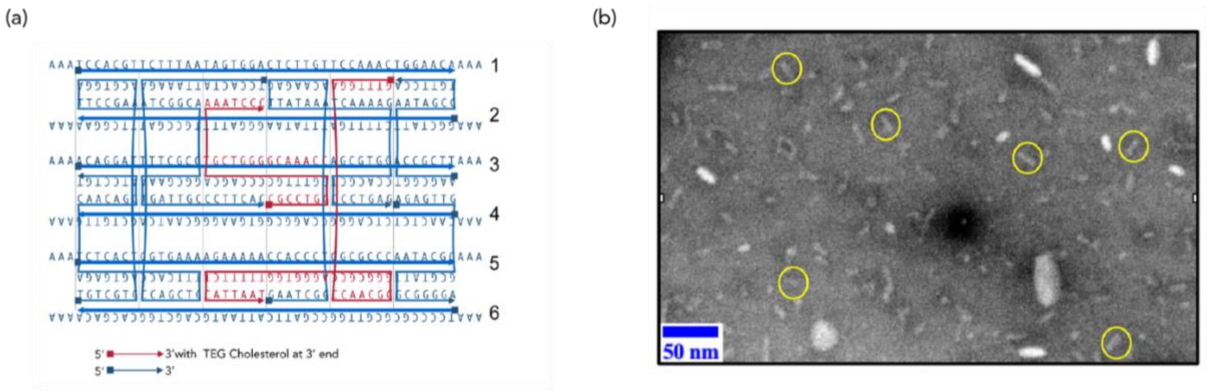

Supplementary Fig. 2 6HB-2C nanopore design, conformation and simulations. (a) Sequence design and strand crossover details. Red strands indicate those oligos that have been modified at the 3' end with Tri-ethylene Glycol (TEG) cholesterol moieties. Blues strands indicate oligos without any modifications. Squares indicate the 5' end while triangles indicate the 3' end of DNA. (b) Negatively stained TEM micrograph of the 6HB nano-barrels. Yellow circles show the nanopores in a flat orientation.

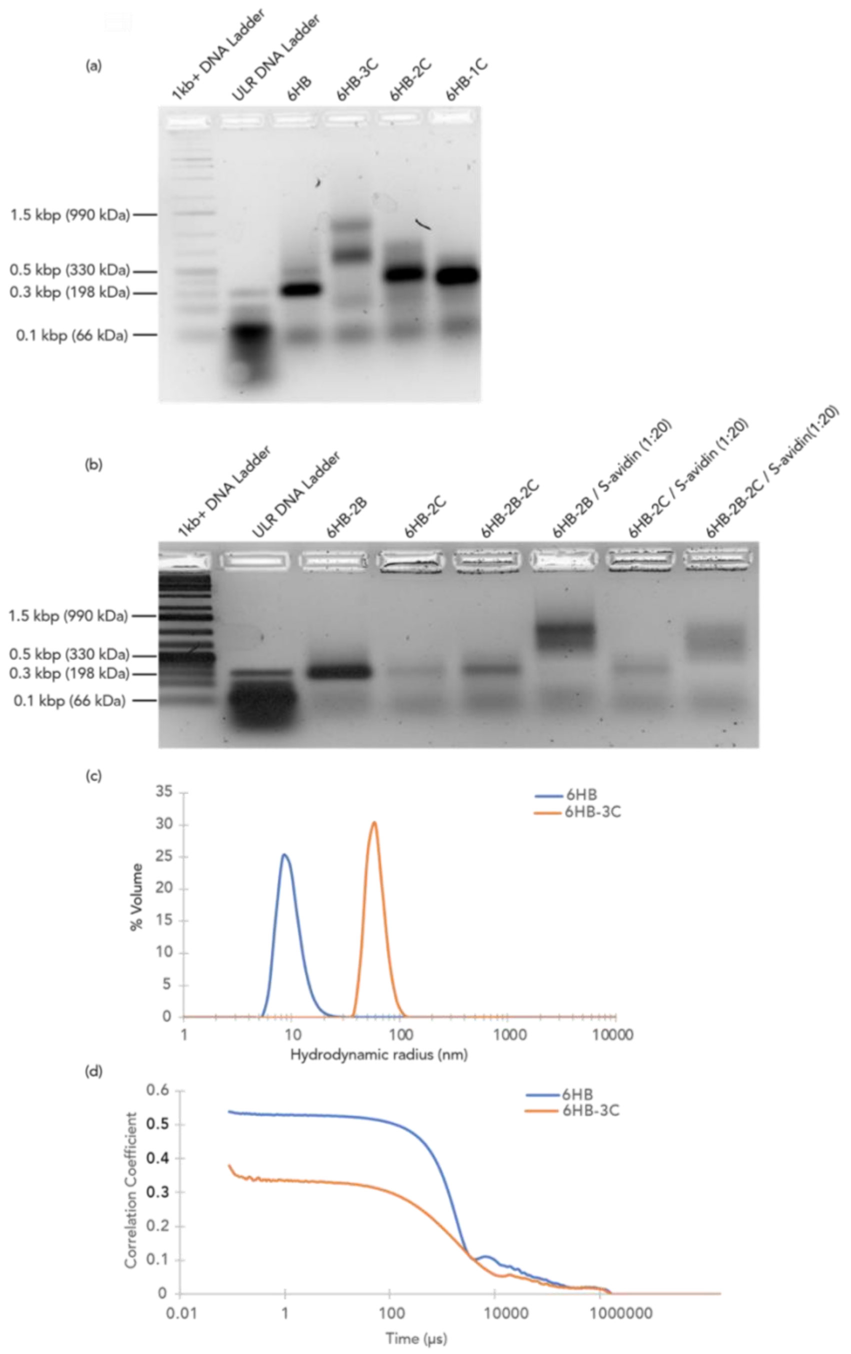

Supplementary Fig. 3 Design and verification of DNA nanopores with cholesterol handles. (a) Electrophoresis characterization (2% Agarose gel) of the nanopores with different number of cholesterol tags around the midsection of the nano-barrel. Lane 1 and 2, DNA ladders; lane 3, fluorescent 6HB nanopores without cholesterol tags; lane 4, 6HB-3C fluorescent nanopores with three cholesterol tags; lane 5, 6HB-2C fluorescent nanopores with two cholesterol tags; lane 6,

6HB-1C fluorescent nanopores with one cholesterol tag. The position of the Kilobase pair length of dsDNA markers is indicated on the left of the gel. (b) Electrophoresis characterization (2% Agarose gel) of migration patterns of biotin modified nanopores in presence and absence of excess Streptavidin. Lanes 1 and 2, DNA ladders; lane 3, 6HB-2B nanopores without cholesterol modifications; lane 4, 6HB-2C nanopores without biotin modifications; lane 5, 6HB-2B-2C modifications; lane 6, 6HB-2B modifications with excess streptavidin (1x: 20x); lane 7, 6HB-2C modifications with excess streptavidin (1x: 20x); lane 8, 6HB-2C modifications with excess streptavidin (1x: 20x). The position of the base pair length of dsDNA markers are indicated in blue on the migration bands. (c) Dynamic light scattering trace of volume-based size distribution of 6HB nanopores (blue line) without any cholesterol tags and 6HB-3C nanopores (orange line) containing three cholesterol tags as measured on Malvern zetasizer instrument. This experiment was repeated 5 times for each sample and the results shown are the average values. (d) Size correlograms for 6HB and 6HB-3C nanopores showing the raw correlation function versus delay time data in the form of $G2(\tau) - 1$. Both the samples show multiple scattering with intercepts less than 1 owing to that fact that the intensity-based size calculations include large scattering effects from multiple size populations and aggregates present even in extremely low fractions. This experiment was repeated 5 times for each sample and the results shown are the average values.

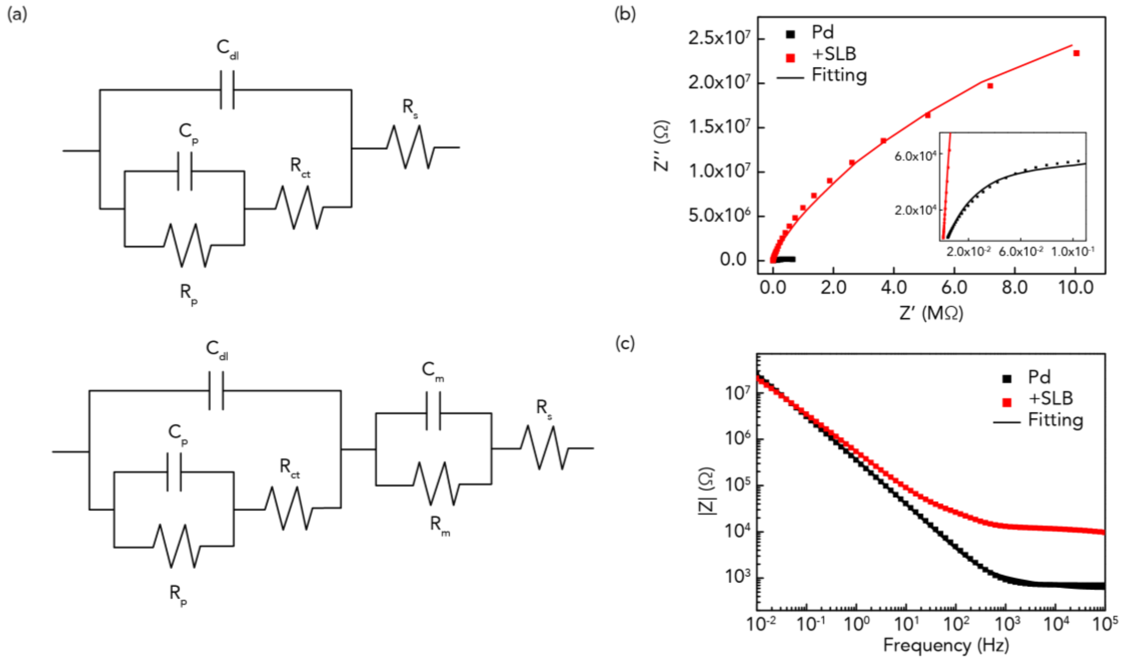

Supplementary Fig. 4 EIS measurement of bioprotonic device and lipid bilayer. (a) Equivalent circuit schematic utilized to fit experimental data. (Top) Bioprotonic device, (Bottom) Bioprotonic device with SLB. The electrolyte solution resistance, Rs, in series with membrane capacitance, Cm, membrane resistance, Rm, double layer capacitance, C_{dl}, charge transfer resistance, R_{ct}, adsorption resistance R_p, and adsorption capacitance. The fitting to the experimental data was performed using the ZSimpWin software, and the results are provided in Supplementary Table 1. (b) Nyquist plot illustrating the relationship between the real and imaginary part of the impedance for both bioprotonic devices and lipid bilayers. (c) Bode plot depicting the magnitude and phase of impedance as a function of frequency (Black: Pd and Red: SLB).

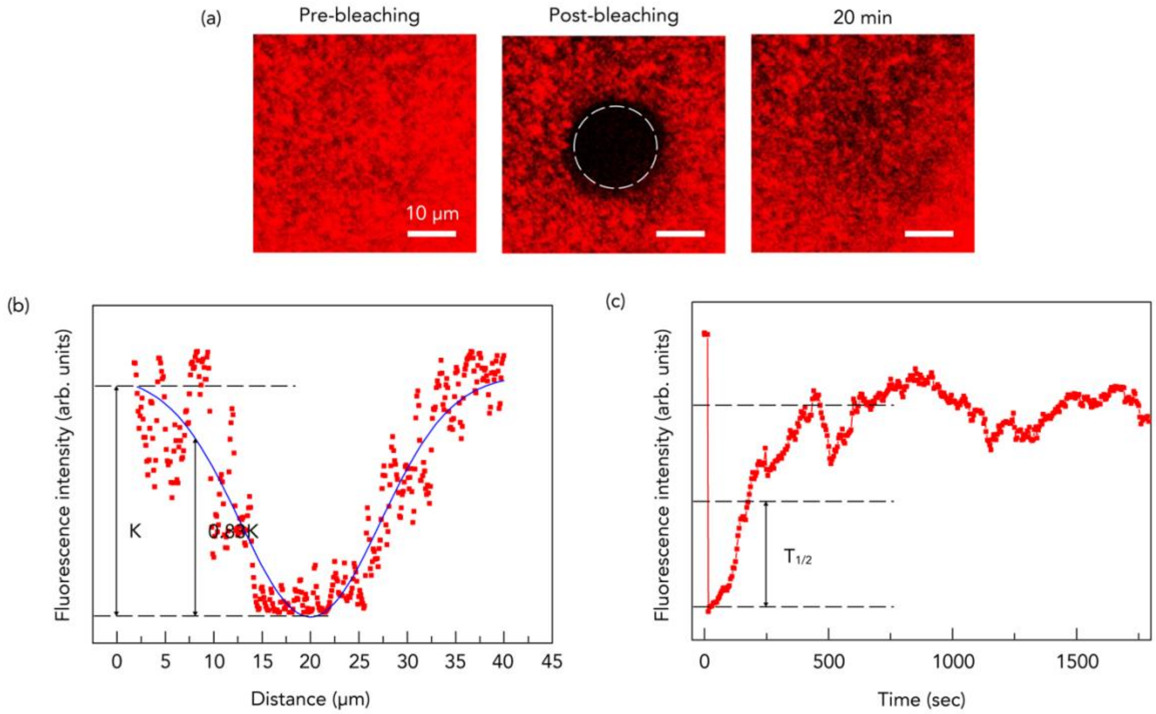

Supplementary Fig. 5 Characterization of lipid bilayer formation on Pd by FRAP. (a) Fluorescence intensity recovery after photobleaching is shown at t = pre, 0, and 20 min (from left to right) (b) Determination of effective bleaching spot. The Gaussian amplitude function was used to extract the effective bleaching spot radius. (c) The normalized fluorescence intensity of FRAP recovery curve. This experiment was independently repeated three times, and the results shown here are representative.

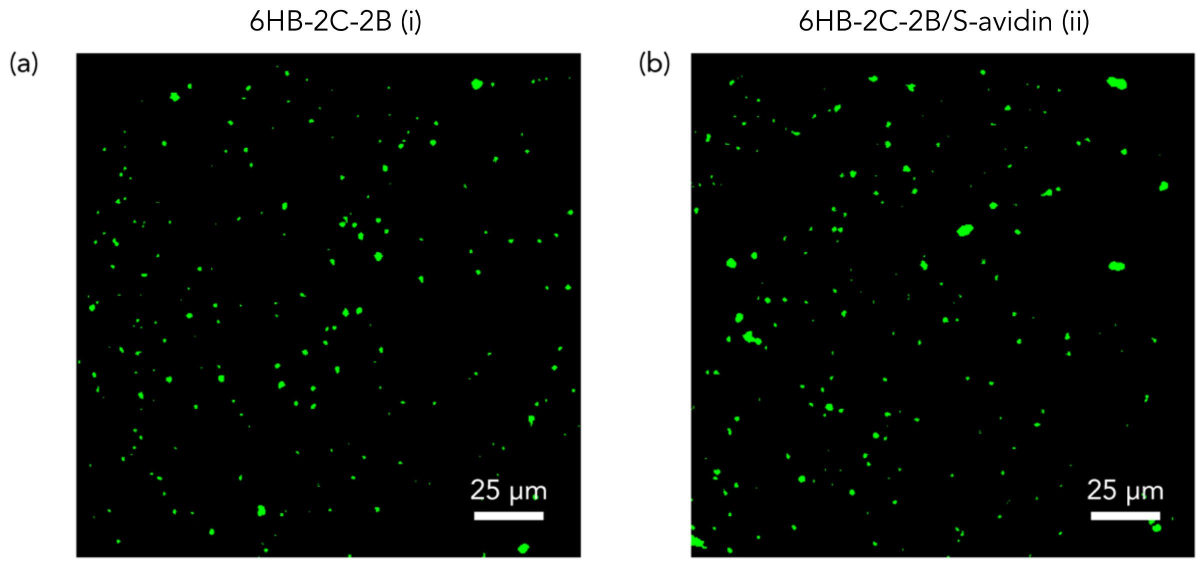

Supplementary Fig. 6 Fluorescence images of 2-cholesterol handled DNA nanopores before and after S-avidin binding. (a) Fluorescence image of DNA nanopores with 2-cholesterol and biotin in the absence of streptavidin (6HB-2C-2B), showing ~205 nanopores. (b) Fluorescence image of the same nanopores after binding with S-avidin (6HB-2C-2B/S-avidin), showing ~200 nanopores. This experiment was independently repeated five times, and the results shown here are representative.

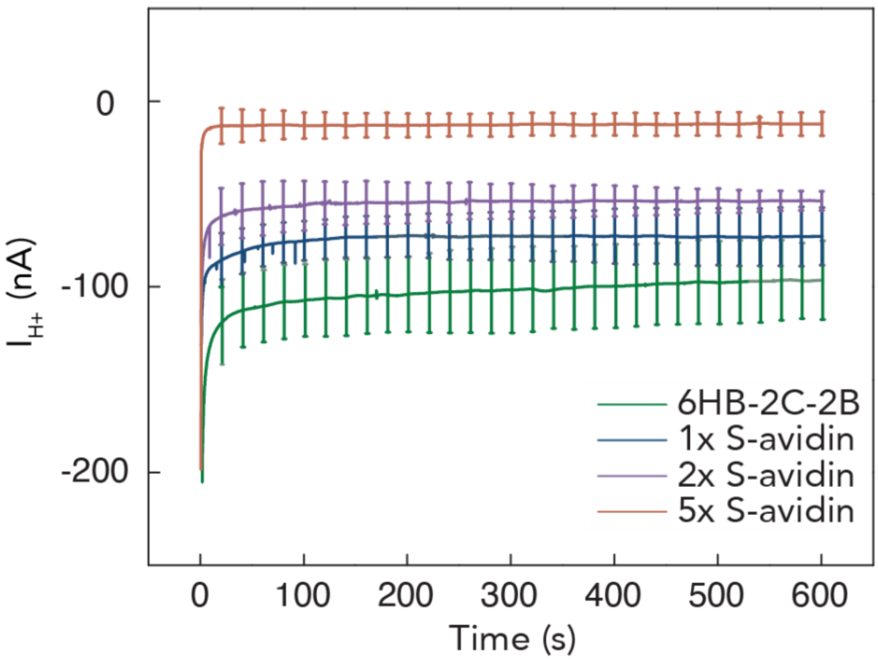

Supplementary Fig. 7 I_{H+} versus time plot for V = -400 mV under different concentrations of S-avidin. Green trace 6HB-2C-2B, blue trace 6HB-2C-2B/1X S-avidin (30 nM), purple trace 6HB-2C-2B/2X S-avidin (60 nM) and red trace 6HB-2C-2B/5X S-avidin (150 nM) which is also the same with the 6HB-2C-2B/S-avidin in Fig. 3b. We measured I_{H+} = -96 ± 21 nA, -73 ± 16 nA, -54 ± 5 nA and -12 ± 6 nA with DNA_Biotin (6HB-2C-2B), 6HB-2C-2B/1X S-avidin, 6HB-2C-2B/2X S-avidin, and 6HB-2C-2B/5X S-avidin respectively. Error bars are 1 s.d. (*n* = 3). Obviously, the increase of S-avidin's concentration leads to the decrease of I_{H+}.

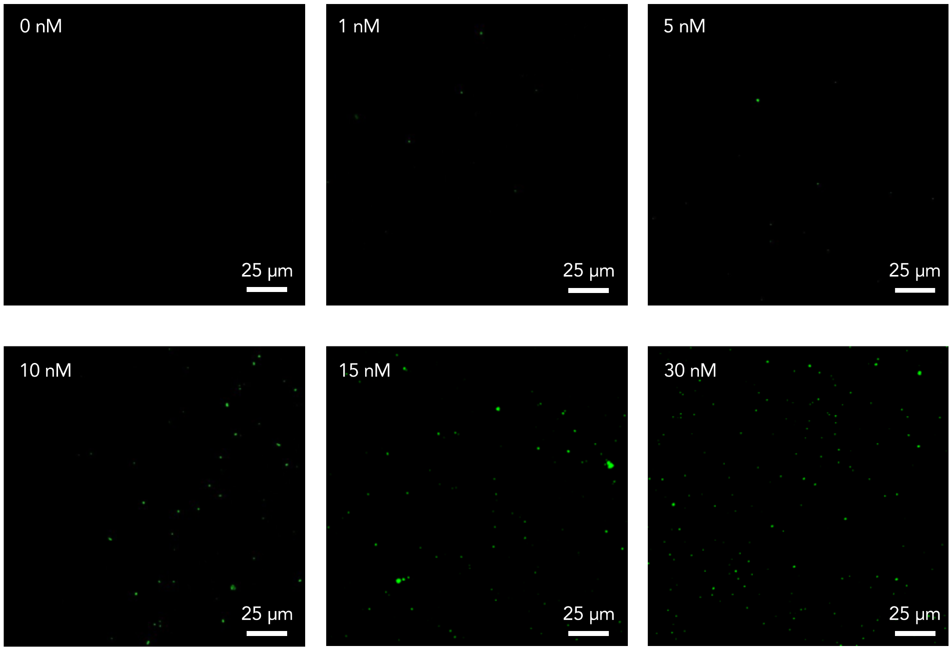

Supplementary Fig. 8 Fluorescence images of DNA nanopores at different concentrations. This experiment was independently repeated five times, and the results shown here are representative.

Supplementary Table 1. Fitted parameter for equivalent circuit model of the bioprotonic device and lipid bilayer

	R_s (Ω)	C_m (F)	R_m (Ω)	CPE_{dl} (F)	R_{ct} (Ω)	CPE_p (F)	R_p (Ω)
Bare Pd	753.9			8.082e-8	2.09e5	1.328e-7	4.551e7
Pd/SLB	753.4	6.959e-6	3.953e-6	1.15e-7	4.784e4	8.919e-7	1.097e6

The overall impedance of lipid bilayer with bioprotonic device was found to be higher than that of the bare device, as evidenced by the larger semicircle in the Nyquist plot. However, in the table, the charge transfer resistance (R_{ct}) values were similar between the two systems. This discrepancy can be attributed to the presence of a gap between the lipid bilayer and the device surface. It may result in an additional resistance component, the membrane resistance (R_m), which contributes to the overall impedance of the lipid bilayer with bioprotonic devices. In addition, the Rm value obtained in this study is similar to the reported in the reference paper, suggesting that the lipid bilayer does not significantly impede the charge transfer process of the device surface but introduces an additional resistive element.

Supplementary Table 2. Polydispersity Index values for dynamic light scattering data

**Name**	**6HB**	**6HB-3C**
Z-Average (nm)	138.8	1097
Polydispersity Index (PI)	0.8249	0.7364
Peak One Width by Number (nm)	2.636	12.25
Peak One Mean by Number (nm)	9.755	60.48
Peak Two Width by Number (nm)	-	165.3
Peak Two Mean by Number (nm)	-	683.2
Intercept	0.7152	0.5497
In Range (%)	90.4	81.02
Fit Error	0.03045	0.00771

Intensity based Polydispersity index (PI) and hydrodynamic Z-average size (cumulants mean) values for 6HB and 6HB-3C nanopores averaged over 5 scans as observed on Malvern zetasizer instrument. Since a small percentage of aggregates are expected and can heavily skew the calculations owing to large scattering effects, number based mean values are used and contrasted against the intensity based-calculations to provide a more relevant estimate of the nanopore population distribution.
