# Supplementary material for: DNA nanopores as artificial membrane channels for bioprotonics
Source: Nat Commun. 2023 Sep 4;14:5364. doi: 10.1038/s41467-023-40870-1 (PMC10477224; doi:10.1038/s41467-023-40870-1)
Supplement: Supplementary file 3 — Description of Additional Supplementary Files [file 41467_2023_40870_MOESM3_ESM.pdf]

Description of Additional Supplementary Files

1. File Name: Supplementary Data 1
   Legend: DNA oligo sequences
